# Supplementary material for: Protein engineering using variational free energy approximation
Source: Nat Commun. 2024 Dec 1;15:10447. doi: 10.1038/s41467-024-54814-w (PMC11609274; doi:10.1038/s41467-024-54814-w)
Supplement: Supplementary file 1 — Supplementary Information [file 41467_2024_54814_MOESM1_ESM.pdf]

Supplementary materials: Protein engineering using variational free energy approximation

Evgenii Lobzaev[1,2], Michael A. Herrera[1], Martyna Kasprzyk[1], and Giovanni Stracquadanio[1,*]

[1]School of Biological Sciences, The University of Edinburgh, Edinburgh EH9 3BF, United Kingdom

[2]School of Informatics, The University of Edinburgh, Edinburgh EH8 9AB, United Kingdom

[*]Corresponding author. Email: giovanni.stracquadanio@ed.ac.uk.

List of Figures

1 **Abridged bacterial L-arginine biosynthetic pathway.** 3

2 **PRotein Engineering by Variational frEe eNergy approximaTion (PREVENT).** 4

3 **Test set for model evaluation.** 4

4 **Commercial *E. coli* BW25113 △argB cured using Flp-FRT recombination.** 5

5 **Construction and expression of pKCHU-argB.** 6

6 **Nucleotide alignment of native and re-coded *E. coli argB* coding sequences.** . . 7

7 **Performance of BW25113 △*argB* transformed using native or re-coded pKCHU-*argB* expression constructs.** 8

8 **Robust *E. coli* transformation using an Opentrons OT-2 robot.** 9

9 **Exemplar transformation plate of *Ec*NAGK variants, demonstrating the array of library transformation efficiency.** 9

10 ***Ec*NAGK hydropathy plot with top performing candidates mutations.** 10

11 **Conservation for Gly123.** 10

12 **RT-qPCR of the top performing candidates from each category.** 11

13 **Uncropped scan of colony PCR (Supplementary Figure 5B).** 11

14 **Uncropped scan of SDS-PAGE analysis (Supplementary Figure 5C).** 12

List of Tables

1 **Models performance on test set.** 13

2 **Hyperparameter analysis on test set.** 14

3 **Hyperparameter analysis on 40 designed *Ec*NAGK variants.** 14

4 **Basic parts used for the creation of pKCHU-*argB*.** 14

5 **Sequencing primers used for pKCHU-*argB* expression constructs (wildtype and variant).** 15

6 **RT-qPCR primers used for pKCHU-*argB* expression constructs (native wildtype, re-coded wildtype and variants) and *rrsA*.** 15

7 **Lag phase duration for selected variants.** 15

Supplementary Figures

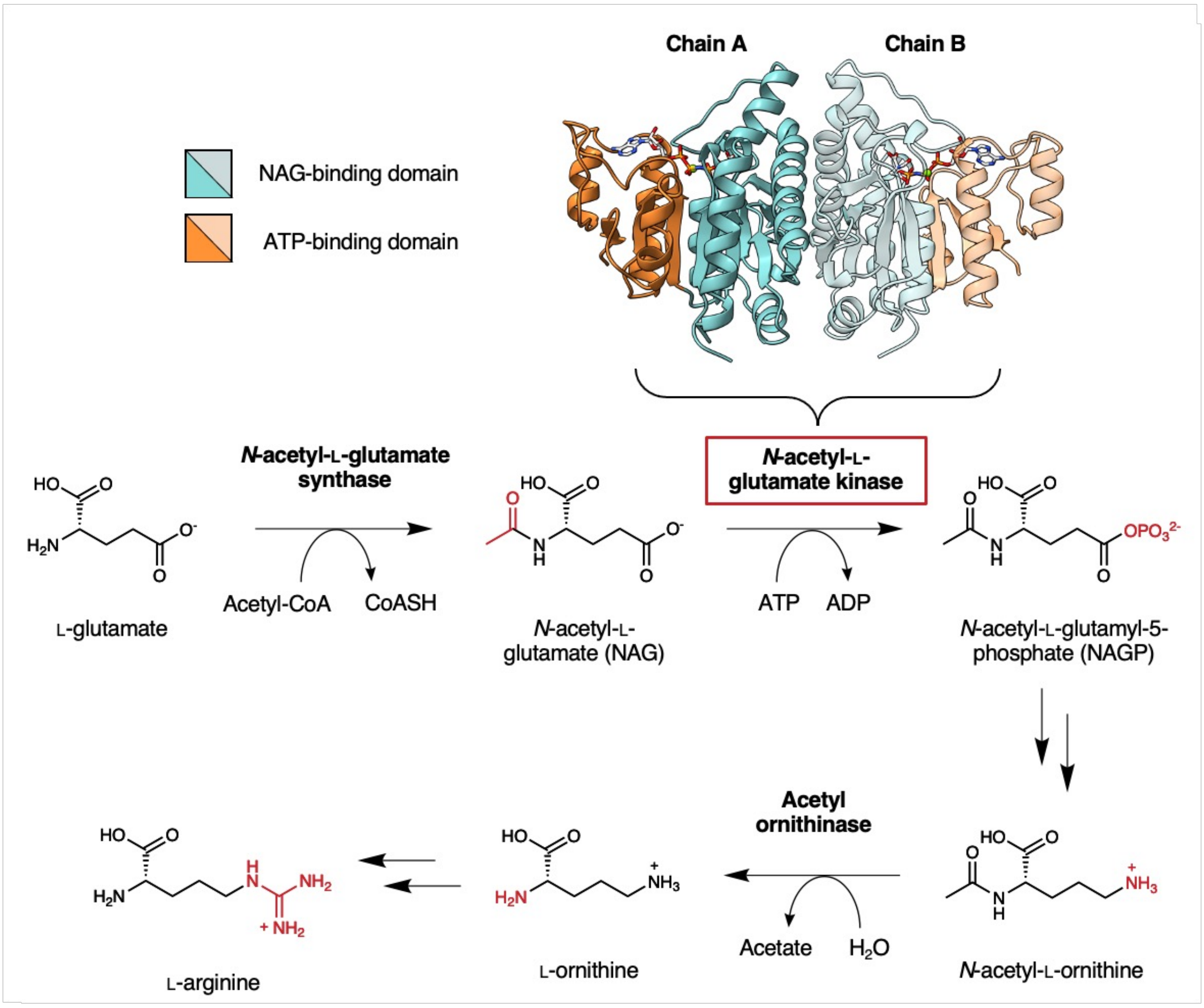

Supplementary Figure 1: Abridged bacterial L-arginine biosynthetic pathway. The crystal structure of the target *E. coli* N-acetyl-L-glutamate kinase (*Ec*NAGK) (PDB: 1GS5) is shown.

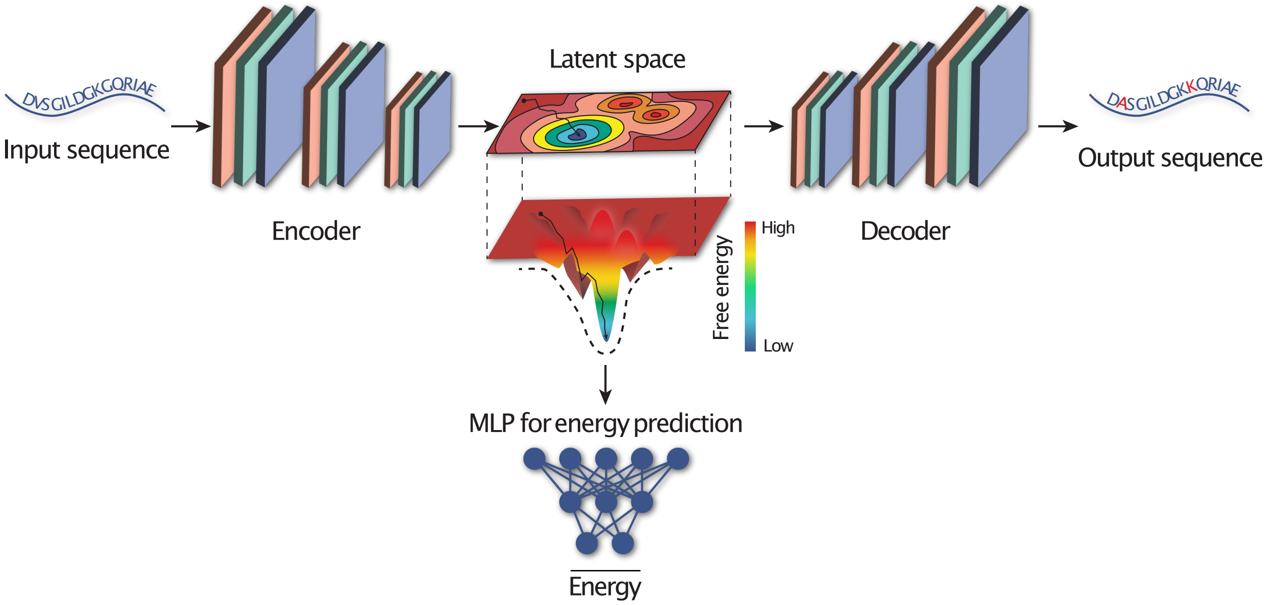

Supplementary Figure 2: PRotein Engineering by Variational frEe eNergy approximaTion (PREVENT). The model takes in input protein sequences and associated free energy values and uses a transformer encoder to map this information to a latent Gaussian space. Samples from the latent space are then sampled and decoded by transformer decoder, to obtain an amino acid sequence, and a multi-layer perceptron to obtain the expected free energy value.

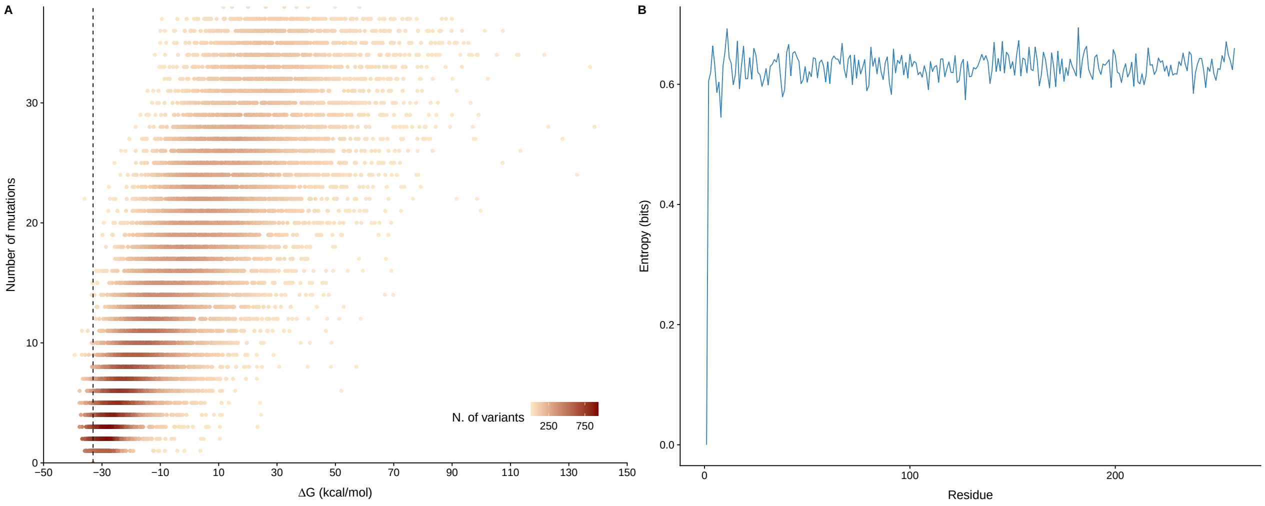

Supplementary Figure 3: Test set for model evaluation. A) Thermodynamic landscape approximation (ΔG) as a function of the number of mutations in *Ec*NAGK variants in the test dataset. Black dashed line denotes the free energy of the wildtype *Ec*NAGK. B) Amino acid entropy of *Ec*NAGK variants in the test dataset. Every position, except the first methionine, is mutated in 6.58% of the generated variant.

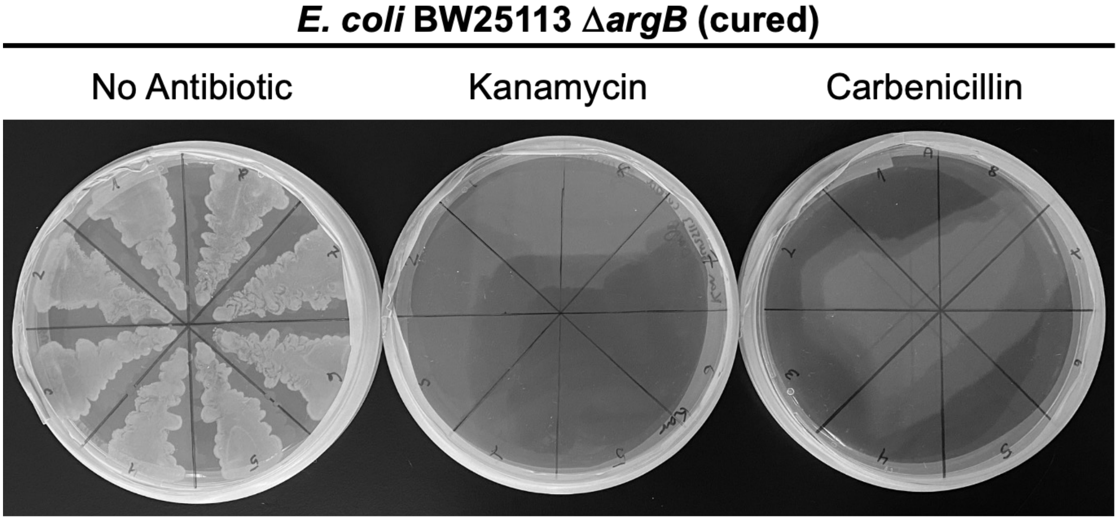

Supplementary Figure 4: Commercial *E. coli* BW25113 △argB cured using Flp-FRT recombination. Eight putatively cured colonies were streaked on selective and nonselective YEP-agar plates to test their re-engineered susceptibility to antibiotics.

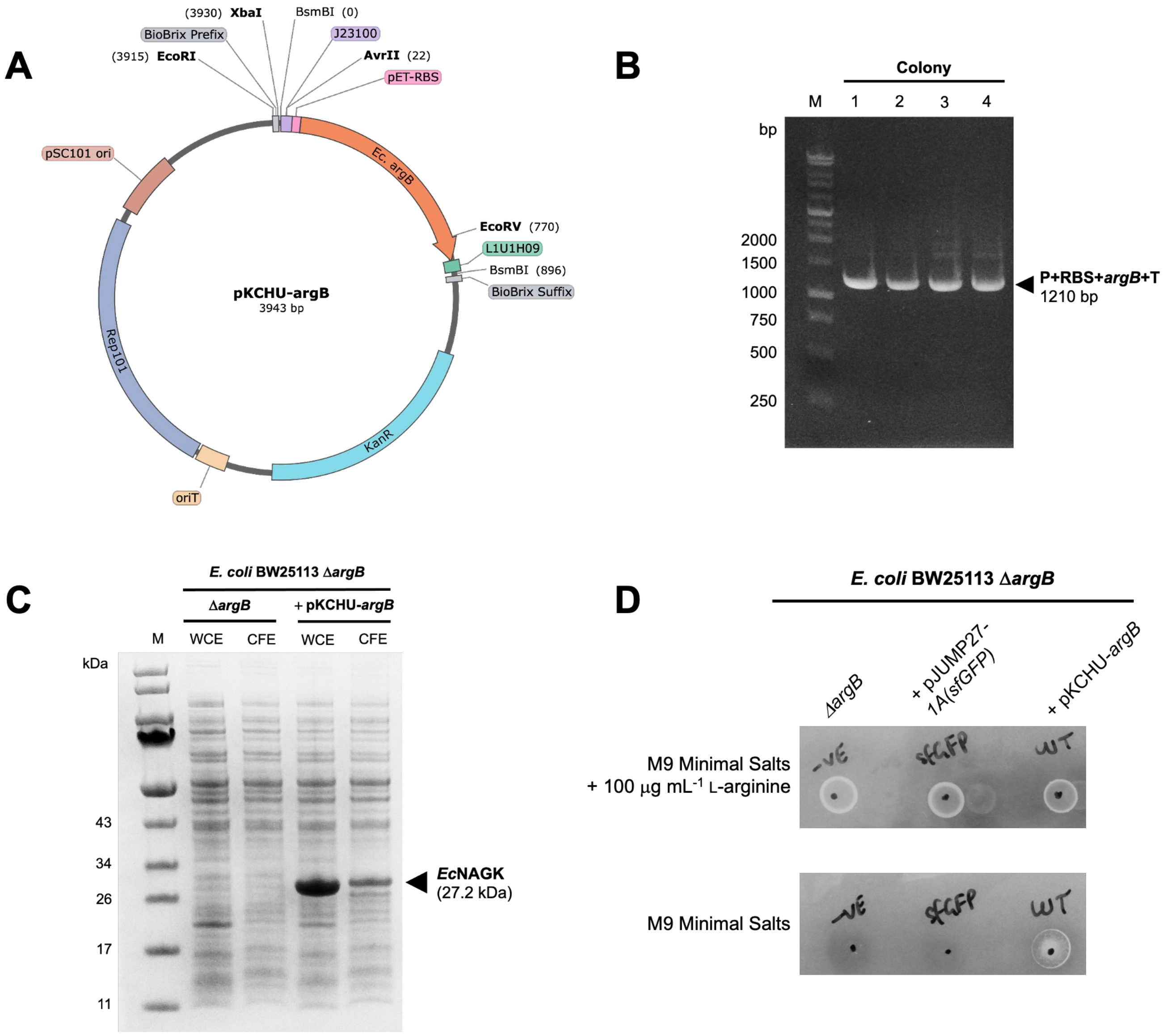

Supplementary Figure 5: Construction and expression of pKCHU-argB. A) Plasmid map highlighting key features of pKCHU-argB including the promoter (J23100), RBS (pET-RBS) and terminator (L1U1H09). pJUMP27-1A(*sfGFP*) was selected as the destination vector for assembly. B) Colony PCR of NEB5-alpha transformants following the level 0 JUMP assembly of pKCHU-argB. Backbone-specific primers provided complete coverage of the assembled promoter, RBS, CDS and terminator (1210 bp). C) SDS-PAGE analysis of BW25113 ΔargB cultures with and without pKCHU-argB. Whole-cell extracts (WCE) show the total protein content (soluble and insoluble) of the biomass sample. Cell-free extracts (CFE) show soluble protein content of the biomass sample following non-mechanical lysis and lysate clarification. D) Auxotrophic selection of pKCHU-argB transformants on M9 salts minimal media after 48 hours of incubation. Both untransformed and pJUMP27-1A(*sfGFP*)-transformed cells were used as negative controls. A positive control plate containing supplemental L-arginine is also shown.

```
               1        10        20        30        40        50        60
argB_Native    ATGATGAATCCATTAATTATCAAACTGGGCGGCGTACTGCTGGATAGTGAAGAGGCGCTG
argB_Re-coded  ATGATGAACCCGCTGATCATCAAACTGGGTGGTGTTCTGCTGGACTCTGAAGAAGCTCTG

                        70        80        90       100       110       120
argB_Native    GAACGTCTGTTTAGCGCACTGGTGAATTATCGTGAGTCACATCAGCGTCCGCTGGTGATT
argB_Re-coded  GAACGTCTGTTCTCTGCTCTGGTTAACTACCGTGAATCTCACCAGCGTCCGCTGGTTATC

                       130       140       150       160       170       180
argB_Native    GTGCACGGCGGCGGTTGCGTGGTGGATGAGCTGATGAAAGGGCTGAATCTGCCGGTGAAA
argB_Re-coded  GTTCACGGTGGTGGTTGCGTTGTTGACGAACTGATGAAAGGTCTGAACCTGCCGGTTAAA

                       190       200       210       220       230       240
argB_Native    AAGAAAAACGGCCTGCGGGTGACGCCTGCTGATCAGATAGACATTATCACCGGAGCACTG
argB_Re-coded  AAAAAAAACGGTCTGCGTGTTACCCCGGCTGACCAGATCGACATCATCACCGGTGCTCTG

                       250       260       270       280       290       300
argB_Native    GCGGGAACGGCAAATAAAACCCTGTTGGCATGGGCGAAGAAACATCAGATTGCGGCCGTA
argB_Re-coded  GCTGGTACCGCTAACAAAACCCTGCTGGCTTGGGCTAAAAAACACCAGATCGCTGCTGTT

                       310       320       330       340       350       360
argB_Native    GGTTTGTTTCTCGGTGACGGCGACAGCGTCAAAGTGACCCAGCTTGATGAAGAGTTAGGT
argB_Re-coded  GGTCTGTTCCTGGGTGACGGTGACTCTGTTAAAGTTACCCAGCTGGACGAAGAACTGGGT

                       370       380       390       400       410       420
argB_Native    CATGTTGGACTGGCGCAGCCAGGTTCGCCTAAGCTTATCAACTCCTTGCTGGAGAACGGT
argB_Re-coded  CACGTTGGTCTGGCTCAGCCGGGTTCTCCGAAACTGATCAACTCTCTGCTGGAAAACGGT

                       430       440       450       460       470       480
argB_Native    TATCTGCCGGTGGTCAGCTCCATTGGCGTAACAGACGAAGGGCAACTGATGAACGTCAAT
argB_Re-coded  TACCTGCCGGTTGTTTCTTCTATCGGTGTTACCGACGAAGGTCAGCTGATGAACGTTAAC

                       490       500       510       520       530       540
argB_Native    GCCGACCAGGCGGCAACGGCGCTGGCGGCAACGCTGGGCGCGGATCTGATTTTGCTCTCC
argB_Re-coded  GCTGACCAGGCTGCTACCGCTCTGGCTGCTACCCTGGGTGCTGACCTGATCCTGCTGTCT

                       550       560       570       580       590       600
argB_Native    GACGTCAGCGGCATTCTCGACGGCAAAGGGCAACGCATTGCCGAAATGACCGCCGCGAAA
argB_Re-coded  GACGTTTCTGGTATCCTGGACGGTAAAGGTCAGCGTATCGCTGAAATGACCGCTGCTAAA

                       610       620       630       640       650       660
argB_Native    GCAGAACAACTGATTGAGCAGGGCATTATTACTGACGGCATGATAGTGAAAGTGAACGCG
argB_Re-coded  GCTGAACAGCTGATCGAACAGGGTATCATCACCGACGGTATGATCGTTAAAGTTAACGCT

                       670       680       690       700       710       720
argB_Native    GCGCTGGATGCGGCCCGCACGCTGGGCCGTCCGGTAGATATCGCCTCCTGGCGTCATGCG
argB_Re-coded  GCTCTGGACGCTGCTCGTACCCTGGGTCGTCCGGTTGACATCGCTTCTTGGCGTCACGCT

                       730       740       750       760       770
argB_Native    GAGCAGCTTCCGGCACTGTTTAACGGTATGCCGATGGGTACGCGGATTTAGCTTAA
argB_Re-coded  GAACAGCTGCCGGCTCTGTTCAACGGTATGCCGATGGGTACCCGTATCCTGGCTTAA
```

Supplementary Figure 6: Nucleotide alignment of native and re-coded *E. coli argB* coding sequences. Visualisation was performed using ESpript 3.0.

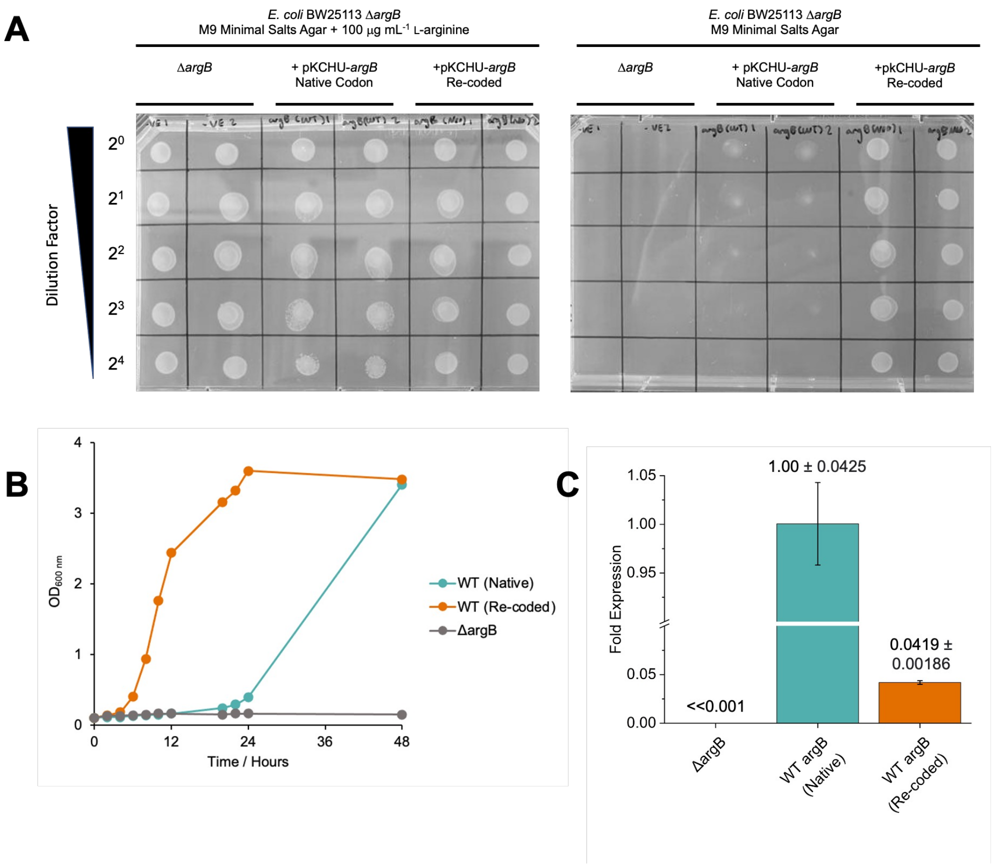

Supplementary Figure 7: Performance of BW25113 ΔargB transformed using native or re-coded pKCHU-*argB* expression constructs. A) Growth discrepancy between pKCHU-*argB* transformants expressing the native or re-coded *argB* on M9 minimal salts agar. A positive control plate containing supplemental L-arginine is also shown. Images were captured after 24 hours of incubation. Experiments were performed in biological duplicate. B) Growth curve comparison of pKCHU-*argB* transformants expressing native or re-coded *argB* in M9 salts minimal media. C) Relative fold-expression of native and re-coded *argB* determined by RT-qPCR. Error bars represent standard deviation of 3 technical replicates.

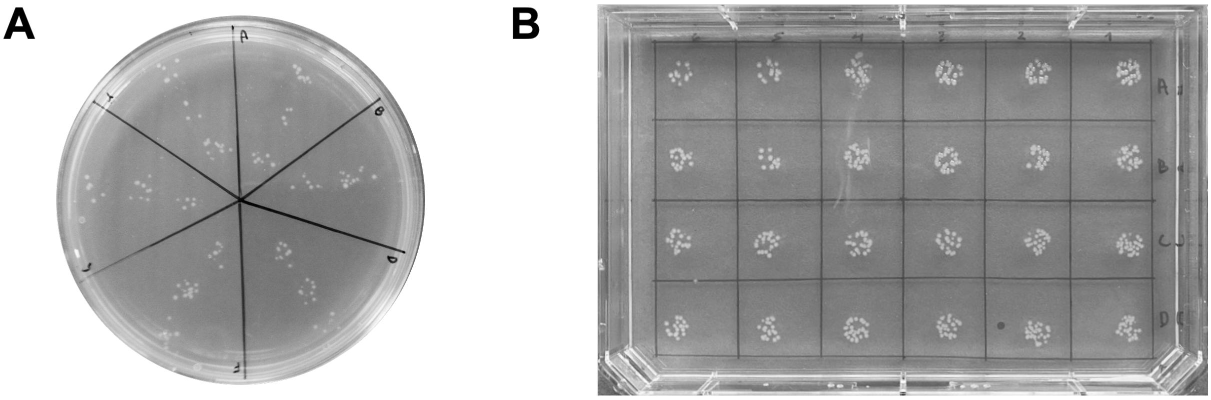

Supplementary Figure 8: Robust *E. coli* transformation using an Opentrons OT-2 robot. A) E. coli DH5α transformed using pKCHU-*argB* and spotted manually on YEP-kanamycin agar. Each segment represents a single biological replicate. B) *E. coli* DH5α transformed using pET23b-*EGFP* and spotted automatically on YEP-carbenicillin agar. Each spot represents a single biological replicate.

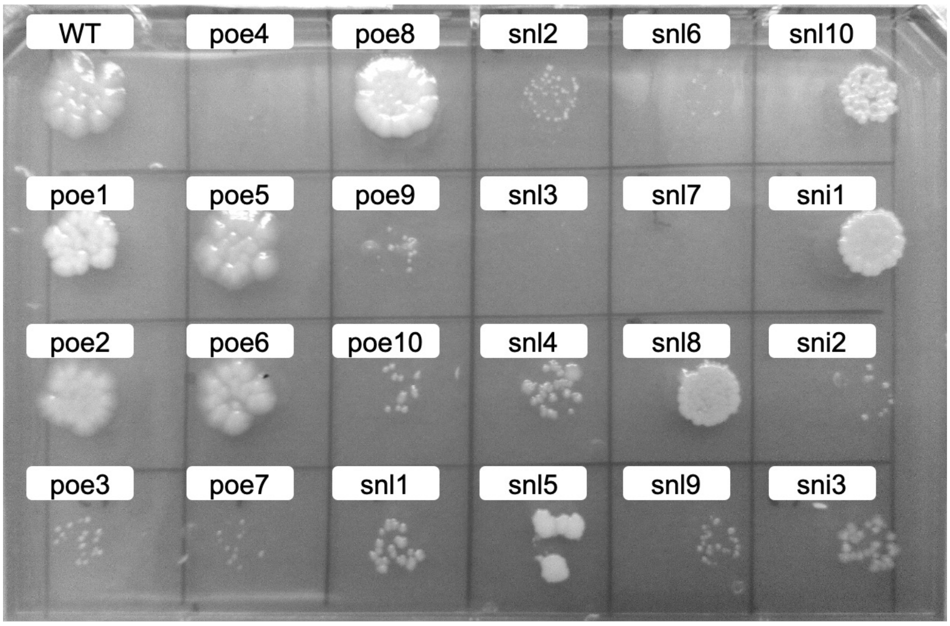

Supplementary Figure 9: Exemplar transformation plate of *Ec*NAGK variants, demonstrating the array of library transformation efficiency. Image was captured after 48 hours of incubation.

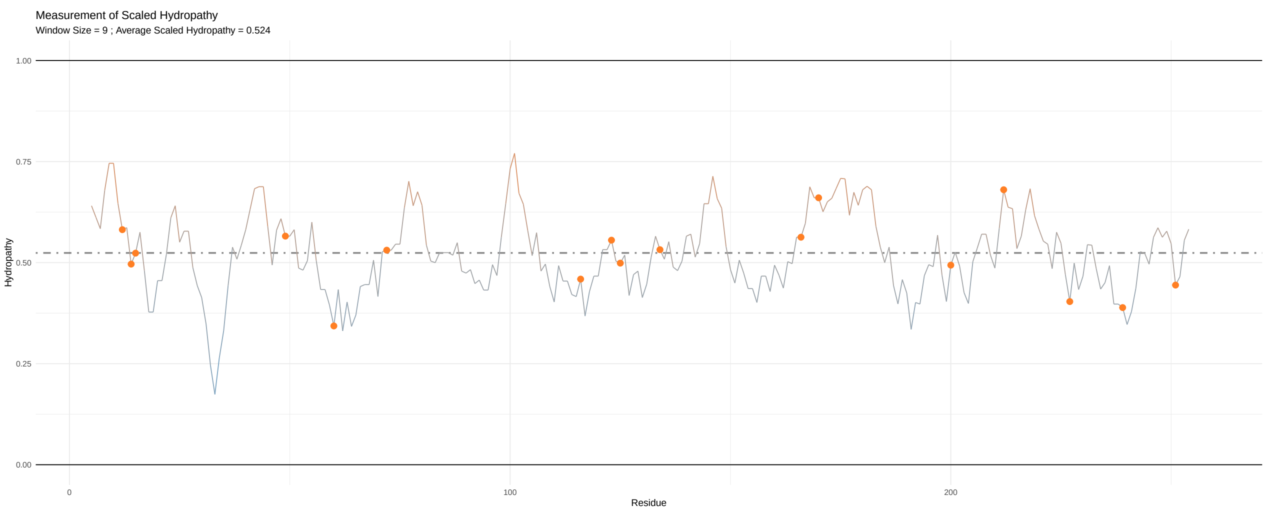

Supplementary Figure 10: *Ec*NAGK hydropathy plot with top performing candidates mutations. Lineplot of the hydropathy index for the wild-type *Ec*NAGK with higher values indicating more hydrophobicity and lower values indicating more hydrophilicity. The dots represent the locations of the mutations in the top performing candidates, namely ”poe1”, ”sne2” and ”snl1”. Variant ”sni1”, with a single mutation in residue 258 is not presented on the plot due to smoothing but the global, non-smoothed, value of the hydropathy index for this residue is 0.7.

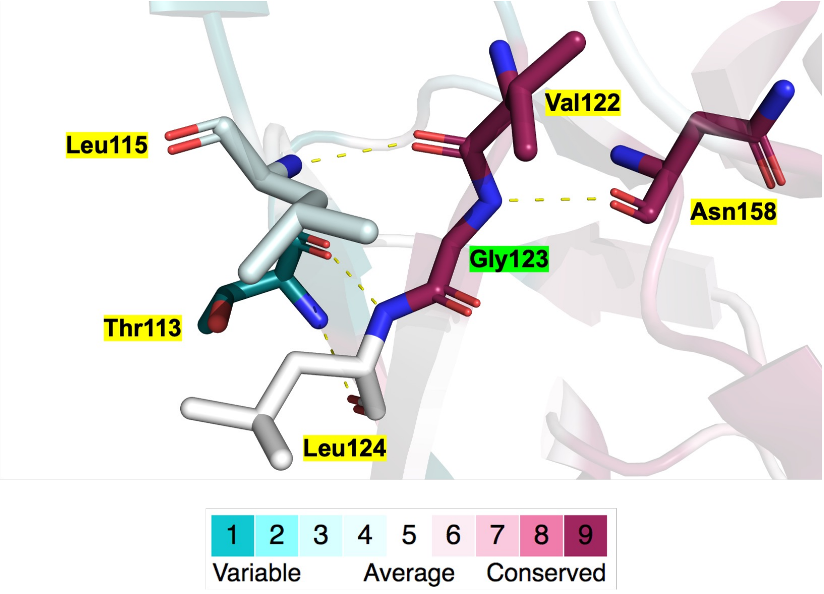

Supplementary Figure 11: Conservation for Gly123. β-sheet interactions between β6, β7 and β10 in wildtype *Ec*NAGK, with evolutionary conservation score mapping (ConSurf). Gly123 is highlighted in green.

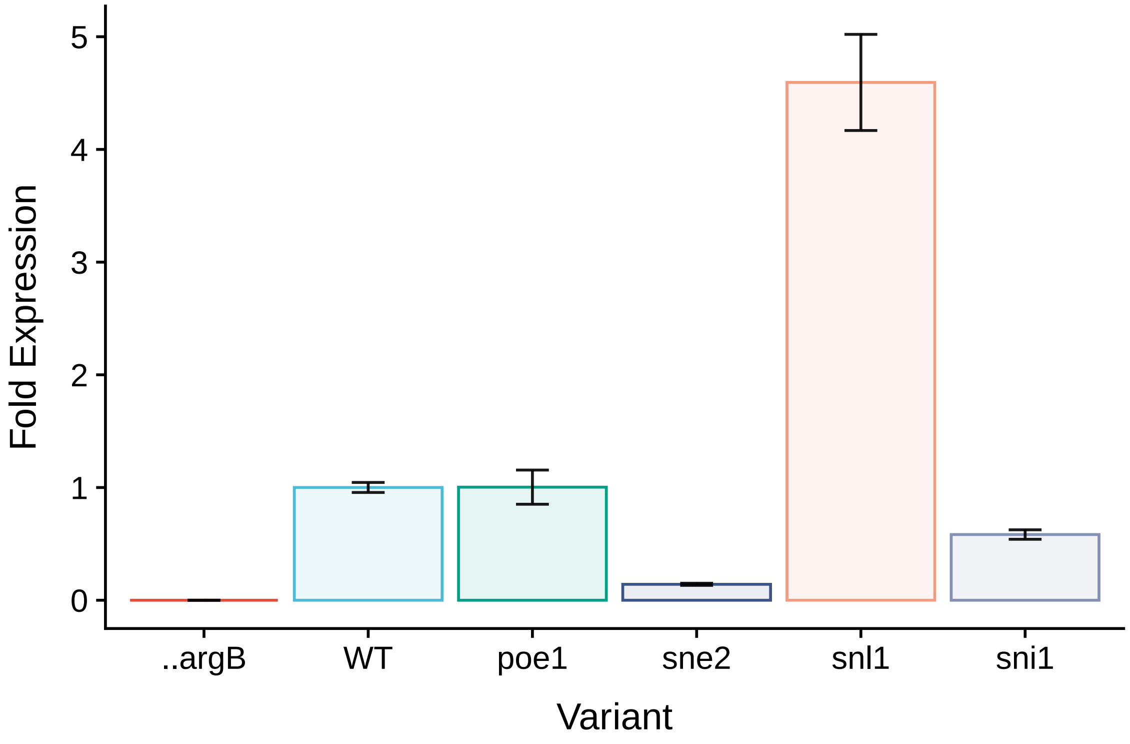

Supplementary Figure 12: RT-qPCR of the top performing candidates from each category.

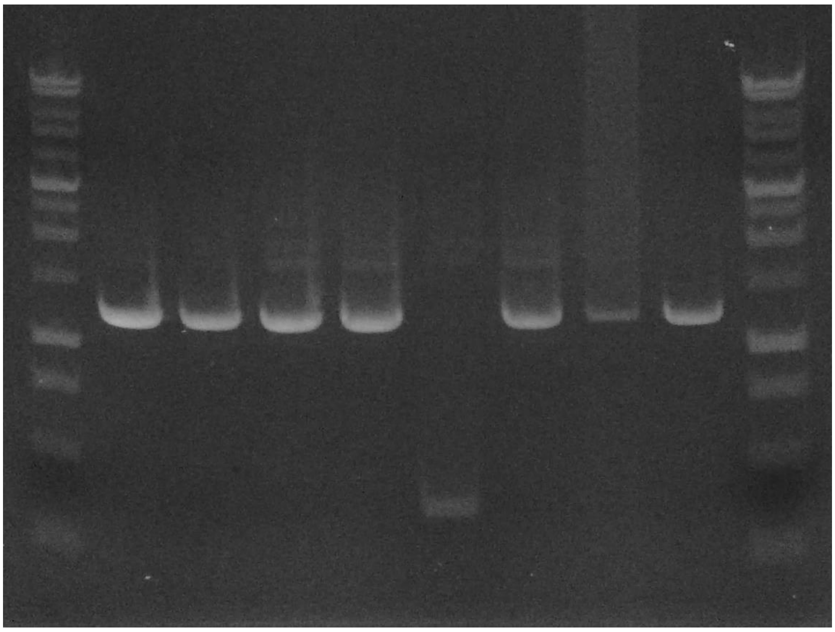

Supplementary Figure 13: Uncropped scan of colony PCR (Supplementary Figure 5B). DNA ladder is in the first column. The rightmost column of is a duplicate of the same DNA ladder.

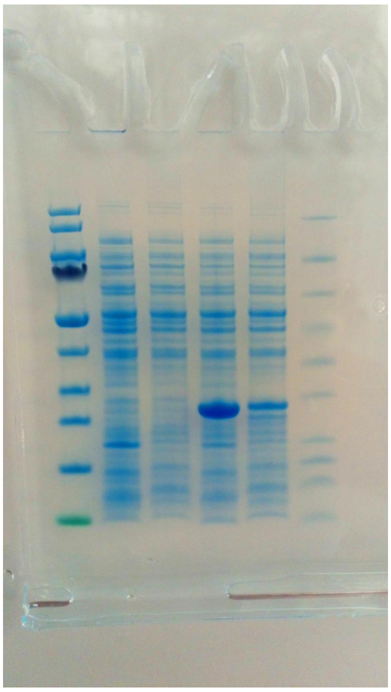

Supplementary Figure 14: Uncropped scan of SDS-PAGE analysis (Supplementary Figure 5C). Molecular weights are in the first column. The rightmost column is an alternative molecular weight ladder that was not suitable for analysis.

Supplementary Tables

Train set size	Reconstruction (PPL)	KL	ELBO	RMSE	Spearman corr
100K	114.80 (0.66)	9.44	124.24	9.27	0.96
75K	115.69 (0.66)	8.94	124.63	9.74	0.95
50K	116.48 (0.66)	8.16	124.64	11.24	0.94
25K	123.42 (0.65)	10.38	133.80	12.12	0.92

Supplementary Table 1: Models performance on test set. For each size of the training set, upon model convergence, we compute average metrics on the test set.

Model	Latent size	Reconstruction	Perplexity (PPL)	KL	ELBO	RMSE	Spearman corr
original	16	110.22	0.67	17.73	127.95	8.42	0.97
	32	110.58	0.67	13.20	123.78	7.13	0.98
	64	109.77	0.67	19.22	129.00	7.61	0.97
	128	110.57	0.67	19.38	129.95	7.61	0.97
small	16	110.93	0.67	17.59	128.52	6.92	0.98
	32	108.42	0.67	19.32	127.74	8.84	0.97
	64	109.49	0.67	16.86	126.35	7.02	0.98
	128	110.20	0.67	16.74	126.94	6.84	0.98

Supplementary Table 2: Hyperparameter analysis on test set. We used our original model (6 encoder layers, 4 decoder layers, 512 embedding size, 8 heads) and a small model (3 encoder layers, 2 decoder layers, 256 embedding size, 4 heads) to evaluate the effects of the latent size and transformer size on the model performance. All models trained for 500 epochs until convergence.

Model	Latent size	Spearman corr	P-value
original	16	0.86	6.68e-13
	32	0.93	3.41e-18
	64	0.90	4.80e-15
	128	0.87	2.30e-13
small	16	0.87	2.13e-13
	32	0.85	5.07e-12
	64	0.90	5.30e-15
	128	0.92	1.57e-17

Supplementary Table 3: Hyperparameter analysis on 40 designed *Ec*NAGK variants. We used our original model (6 encoder layers, 4 decoder layers, 512 embedding size, 8 heads) and a small model (3 encoder layers, 2 decoder layers, 256 embedding size, 4 heads) to compute Spearman correlation between FoldX estimates and predicted energy values. Two-sided t-test was used to compute the p-values.

Part	Part ID	JUMP Part Origin	Description
Promoter	J23100	pJUMP19-J23100_P	Constitutive strong promoter
Ribosome Binding Site	pET-RBS	pJUMP18-RBS-pET_R	pET vector ribosome binding site
Terminator	L1U1H09	pJUMP19-L1U1H09_T	Synthetic terminator
Backbone (destination) vector	pJUMP27-1A(sfGFP)	pJUMP27-1A(sfGFP)	Low copy plasmid with pSC101 origin and super-folder GFP reporter

Supplementary Table 4: Basic parts used for the creation of pKCHU-*argB*.

Primer	JUMP Primer ID	Sequence
Forward	PS1	AGGGCGGCGGATTTGTCC
Reverse	PS2	GCGGCAACCGAGCGTT

Supplementary Table 5: Sequencing primers used for pKCHU-*argB* expression constructs (wildtype and variant).

Target	Primer	Sequence (5' → 3')
rrsA	Forward<br>Reverse	TCCAGGTGTAGCGGTGAAAT<br>TTGAGTTTTAACCTTGCGGC
argB (Native wildtype)	Forward<br>Reverse	AGACGAAGGGCAACTGATGA<br>GCCGCGTTCACTTTCACTAT
argB (Re-coded wildtype + variants)	Forward<br>Reverse	GTCCGCTGGTTATCGTTCAC<br>TAACAGAGTCACCGTCACCC

Supplementary Table 6: RT-qPCR primers used for pKCHU-*argB* expression constructs (native wildtype, re-coded wildtype and variants) and *rrsA*.

Experiment	Lag Phase Duration/Hours	Standard Error	Fold Change
WT	5.64	0.038	1.00
poe1	6.39	0.304	1.13
snl1	6.22	0.159	1.10
sni1	6.55	0.059	1.16
sne2	4.32	0.192	0.77

Supplementary Table 7: Lag phase duration for selected variants.
