# Supplementary material for: Protein engineering using variational free energy approximation
Source: Nat Commun. 2024 Dec 1;15:10447. doi: 10.1038/s41467-024-54814-w (PMC11609274; doi:10.1038/s41467-024-54814-w)
Supplement: Supplementary file 4 — Source Data [file 41467_2024_54814_MOESM4_ESM.zip › source-data/source-data-file.pdf]

Source Data File

Overview

Description of the files and scripts used to generate the figures for the manuscript. All plots for the manuscript are located in the `plots` (used in the main text) and `supplementary-plots` (used in the supplementary information) directories. All scripts generating the plots (and some tables) are located in the `scripts` directory. The `data` and `data-review` directories contain all input data files used in the analysis of the model performance and the designed sequences. The `experimental-results` folder contains the experimental results obtained from the lab experiments with argB protein. The `structures` directory contains the PNG file with the structures of the wildtype and some designed sequences. Finally, `tables` directory contains the tables with the results of the model performance and the designed sequences (used in the supplementary information).

The following R scripts are used to generate the figures in the main text and supplementary information:

- **figure1.R**: generates **Figure 1** from the main text.
- **figure2.R**: generates **Figure 2** from the main text.
- **figure3.R**: generates **Figure 3** from the main text and some supplementary figures, such as **supp-figure-correlations**, which is not used in the current version of the manuscript.
- **figure4-6.R**: generates **Figure 4** - **Figure 6** from the main text.
- **figure7.R**: generates **Figure 7** from the main text and a supplementary figure **supp-figure-12-argb-variant-ct.pdf**, used in the supplementary information as **Supplementary Figure 12**.
- **supp-figure3.R**: generates **Supplementary Figure 3** from the supplementary information (saved as **supp-figure-3-test-set.pdf**).
- **supp-figure10.R**: generates **Supplementary Figure 10** from the supplementary information (saved as **supp-figure-10-hydrophobicity-mutations-window-top-candidates.pdf**).

Additionally, there are 2 jupyter notebooks used to generate tables for the supplementary information:

- **model-performance-analysis.ipynb**: generates **model-performance-test-set.csv**, which is used for **Supplementary Table 1**.
- **model-performance-analysis-hyperparameters.ipynb**: generates **model-performance-test-set-hyperparams.csv** and **variant-pool-hyperparam-results-spearman.csv**, which are used for **Supplementary Table 2** and **Supplementary Table 3** respectively. Additionally it updates **variant-pool.csv** file with results of all models used in hyperparameter analysis and saves it as **variant-pool-extended-with-hyperparam-results-updated.csv**.

Plots

In `plots` folder there are all plots used in the main text. In `supplementary-plots` folder there are all plots used in the supplementary information. The filenames of the plots correspond to the figures'

numbering in the manuscript. The plots are saved in PDF format. The only figure not used in the current version of the manuscript is **supp-figure-correlations.pdf**.

Tables

In `tables` folder there are 4 CSV files, which are used in the supplementary information:

- **model-performance-test-set.csv**: contains the results of the models' performance on the test set and presented in **Supplementary Table 1**.
- **model-performance-test-set-hyperparams.csv**: contains the results of the models' (used in hyperparameter analysis) performance on the test set with different hyperparameters and presented in **Supplementary Table 2**.
- **variant-pool-hyperparam-results-spearman.csv**: contains the results of the Spearman correlation between the ground truth FoldX values and the predicted values for the models used in hyperparameter analysis for 40 designed variants and presented in **Supplementary Table 3**.
- **variant-pool-extended-with-hyperparam-results-updated.csv**: a more detailed version of **variant-pool.csv** file with the results of all models used in hyperparameter analysis.

Experimental results

The `experimental-results` folder contains 2 subfolders:

- `argb-mh-images`: contains various images captured during the lab experiments with argB protein. Most images are used in the supplementary information and copied to the `supplementary-images` folder with renamed filenames.
- `data`: contains CSV and XLSX file with the results of the lab experiments with argB protein.

Structures

The `structures` folder contains the PNG files with the structures of the wildtype and some designed sequences. They are used in **Figures 4-6** in the main text.

Data

The `data` folder contains 4 subfolders with the following data files:

- `generated-samples`: several FASTA and CSV files with the generated sequences by the main model, described in the manuscript. The most important file is **variant-pool.csv**, which contains the detailed information about 40 designed sequences.
- `input-data`: contains the FASTA files with the input data used in the model training and testing (including full and reduced datasets used for training).
- `proton-select-output`: contains the output files for NMA of the designed sequences and IF-scores.
- `trained-models`: contains the result of energy estimation on the test set and 40 designed sequences by 4 models (trained on 25K/50K/75K/100K+ sequences) discussed in the manuscript.

Data Review

The `data-review` folder contains results of energy estimation on the test set and 40 designed sequences by additional 8 models discussed in the manuscript in the hyperparameter analysis section.
