# Supplementary figures and images for: Protein engineering using variational free energy approximation
Source: Nat Commun. 2024 Dec 1;15:10447. doi: 10.1038/s41467-024-54814-w (PMC11609274; doi:10.1038/s41467-024-54814-w)

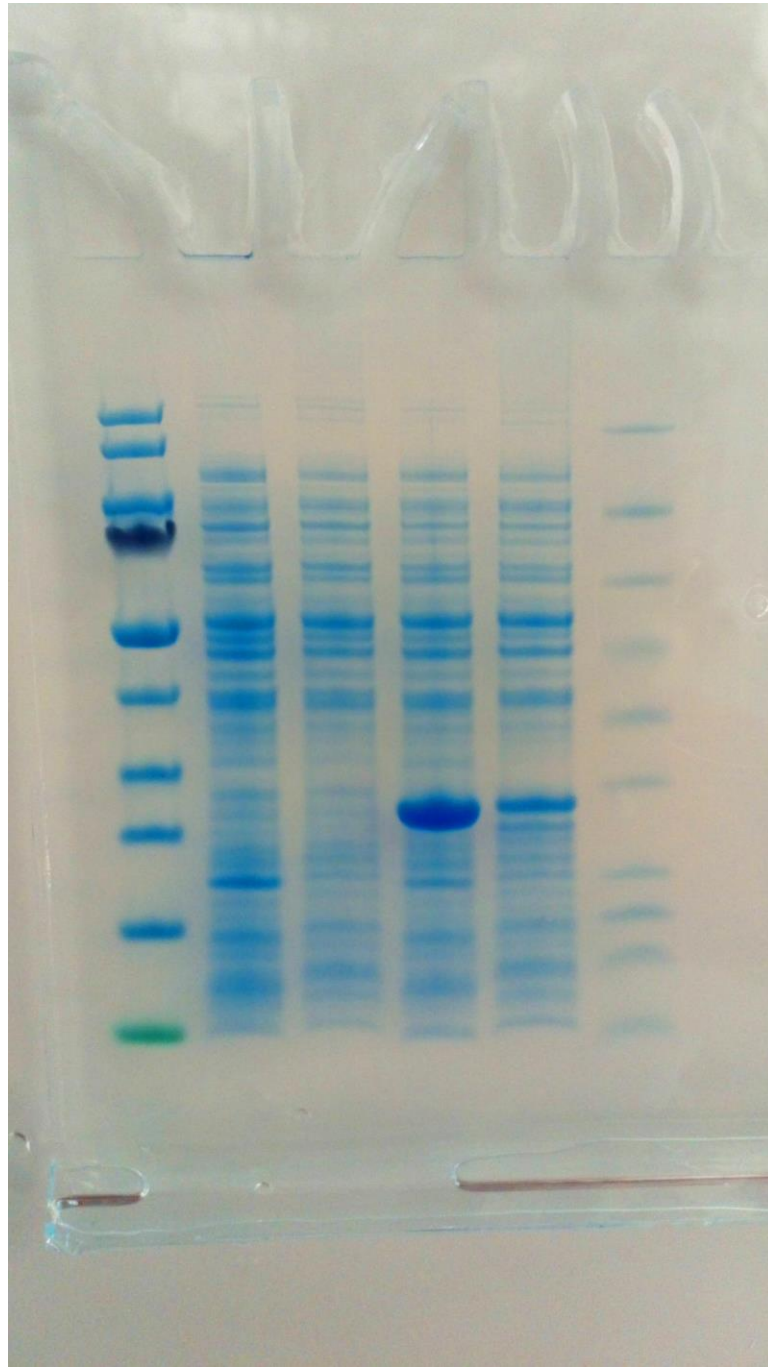

Supplement: Supplementary file 4 — Source Data [file 41467_2024_54814_MOESM4_ESM.zip › source-data/supp-figure-5C-raw.pdf]

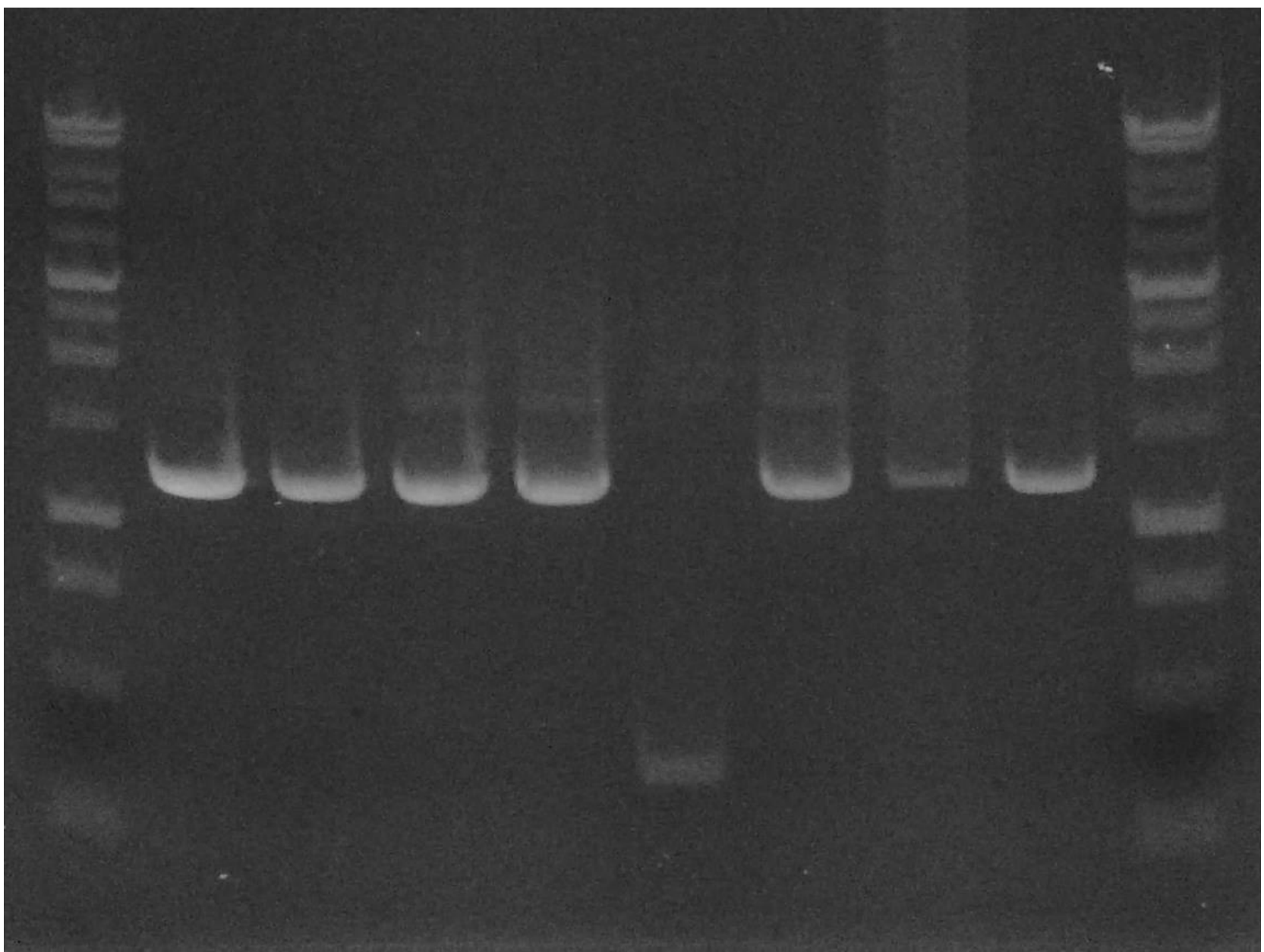

Supplement: Supplementary file 4 — Source Data [file 41467_2024_54814_MOESM4_ESM.zip › source-data/supp-figure-5B-raw.pdf]
